# Apolipoproteins and cancer

**DOI:** 10.1002/cam4.2587

**Published:** 2019-10-01

**Authors:** Liwen Ren, Jie Yi, Wan Li, Xiangjin Zheng, Jinyi Liu, Jinhua Wang, Guanhua Du

**Affiliations:** ^1^ The State Key Laboratory of Bioactive Substance and Function of Natural Medicines Beijing China; ^2^ Key Laboratory of Drug Target Research and Drug Screen Institute of Materia Medica Chinese Academy of Medical Science and Peking Union Medical College Beijing China; ^3^ Department of Clinical Laboratory Peking Union Medical College Hospital Beijing People's Republic of China

**Keywords:** apolipoprotein, autophagy, cancer, drug resistance, oxidative stress

## Abstract

The role of apolipoproteins in cardiovascular disease has been well investigated, but their participation in cancer has only been explored in a few published studies which showed a close link with certain kinds of cancer. In this review, we focused on the function of different kinds of apolipoproteins in cancers, autophagy, oxidative stress, and drug resistance. The potential application of apolipoproteins as biomarkers for cancer diagnosis and prognosis was highlighted, together with an investigation of their potential as drug targets for cancer treatment. Many important roles of apolipoproteins and their mechanisms in cancers were reviewed in detail and future perspectives of apolipoprotein research were discussed.

## Graphical abstract

We focused on roles of different kind apolipoproteins in cancers, autophagy, oxidative stress, resistance of drugs and hypolipidemic agents and cancers. In addition, apolipoproteins as drug targets of cancer treatment and biomarkers for cancer diagnosis and prognosis were discussed. Future perspectives of apolipoproteins research were described in the end.

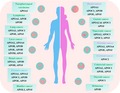

## 1 INTRODUCTION

Apolipoproteins (APOs) bind to lipids to form lipoproteins. By functioning as lipid carriers, apolipoproteins act as ligands for cell membrane receptors, cofactors of enzymes and structural components of lipoproteins.^1^ APOs could bind and transport blood lipids to various tissues of the body for metabolism and utilization. The human apolipoprotein gene family consists of 22 members: APOA1, APOA2, APOA4, APOA5, APOB‐48, APOB‐100, APOC1, APOC2, APOC3, APOC4, APOD, APOE, APOH, APOL1, APOL2, APOL3, APOL4, APOL5, APOL6, APOM, APOO, and APOJ. These 22 apolipoproteins are classified into 10 subfamilies (APOA‐APOJ) (Table 1).

**Table 1:** Information and functions of human apolipoprotein family

Apolipoprotein	Gene	Gene ID	Chromosomal localization	Protein size (amino acids)	Protein MW (Da)	Main function	Affected cancer types
Apolipoprotein A1	APOA1	335	11q23.3	267	30 778	Target, biomarker	NPC, NSCLC, colorectal cancer, lymphoma, prostate cancer, breast cancer, RCC, ovarian cancer
Apolipoprotein A2	APOA2	336	1q23.3	100	11 175	Biomarker	HCC, prostate cancer, gastric cancer, myeloma, pancreatic cancer
Apolipoprotein A4	APOA4	337	11q23.3	396	45 399	Biomarker	HCC, ovarian cancer
Apolipoprotein A5	APOA5	116 519	11q23.3	366	41 213	——	——
Apolipoprotein B	APOB	338	2p24.1	4563	515 605	Biomarker	HCC, bladder cancer, breast cancer,
Apolipoprotein C1	APOC1	341	19q13.32	83	9332	Target, biomarker	Pancreatic cancer, breast cancer, thyroid cancer, prostate cancer, lung cancer, colorectal cancer, gastric cancer
Apolipoprotein C2	APOC2	344	19q13.32	101	11 284	Biomarker	Pancreatic cancer, cervical cancer
Apolipoprotein C3	APOC3	345	11q23.3	99	10 852	——	——
Apolipoprotein C4	APOC4	346	19q13.32	127	14 553	——	——
Apolipoprotein D	APOD	347	3q29	189	21 276	Biomarker	HCC, colorectal cancer, prostate cancer, breast cancer, ovarian cancer, melanoma, RCC
Apolipoprotein E	APOE	348	19q13.32	317	36 154	Target, biomarker	Lung cancer, prostate cancer, HCC, ovarian cancer, gastric cancer, bladder cancer, leukemia, RCC, colorectal cancer, breast cancer
Apolipoprotein H	APOH	350	17q24.2	345	38 298	Target, biomarker	HCC, bladder cancer, renal cancer, leukemia,
Apolipoprotein L1	APOL1	8542	22q12.3	398	43 974	Biomarker	Thyroid cancer
Apolipoprotein L2	APOL2	23 780	22q12.3	337	37 092	Biomarker	Bladder cancer
Apolipoprotein L3	APOL3	80 833	22q12.3	402	44 278	Biomarker	Prostate cancer
Apolipoprotein L4	APOL4	80 832	22q12.3	351	39 164	——	——
Apolipoprotein L5	APOL5	80 831	22q12.3	433	47 044	——	——
Apolipoprotein L6	APOL6	80 830	22q12.3	343	38 128	Target	Colorectal cancer
Apolipoprotein M	APOM	55 937	6p21.33	188	21 253	Target, biomarker	HCC, NSCLC, colorectal cancer
Apolipoprotein O	APOO	79 135	Xp22.11	198	22 285	——	——
Apolipoprotein J	APOJ	1191	8p21.1	449	52 495	Target, biomarker	Prostate cancer, lung cancer, HCC, colon cancer, breast cancer, bladder cancer, RCC, ovarian cancer, gastric cancer, pancreatic cancer

**Figure 1:**
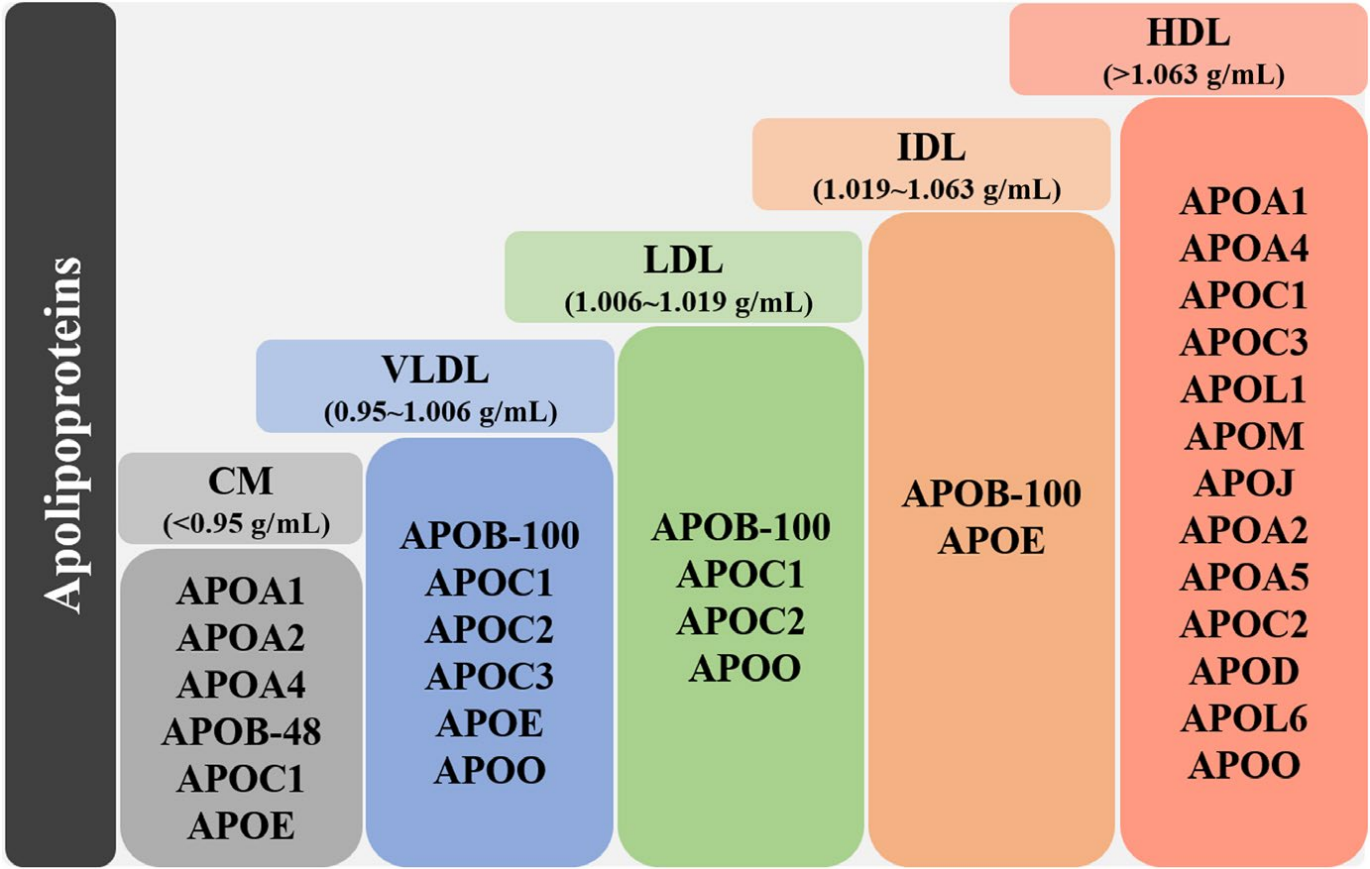
Classification of apolipoproteins. The apolipoproteins are divided into CM, VLDL, LDL, IDL, and HDL types, according to lipoproteins types

Different APOs bind lipids to form lipoproteins of different densities, and lipoproteins can be divided into several types according to their densities (Figure 1): chylomicrons (CM), very low density lipoprotein (VLDL), low density lipoprotein (LDL), intermediate density lipoprotein (IDL), and high density lipoprotein (HDL).^2^ Some APOs can combine with several different types of lipoproteins. For example, APOA1 is the major structural protein component of HDL and it is present in other lipoproteins in smaller amounts. APOB plays a particularly important role in lipoprotein transport being the primary organizing protein of many lipoproteins.^3^

APOs are mainly synthesized in the liver and intestine. In the liver, the synthesis of APOs is affected by alcohol consumption, the administration of lipid‐lowering drugs, fibric acids or niacin, diet and various hormones, estrogens, androgens, insulin, glucagon and thyroxin. In the intestine, the synthesis of apolipoproteins is predominantly controlled by lipid content in the diet.^4^

Evidence from numerous studies has shown that APOs play a vital role in cardiovascular disease, such as atherosclerosis and coronary artery disorders,^1^, ^2^, ^5^ but a number of recent reports have linked apolipoproteins with various types of cancers. Here we review and summarize the current research findings on the function, mechanism, and clinical attributes of all APOs in cancer.

## 2 APOLIPOPROTEINS AND CANCER

**Figure 2:**
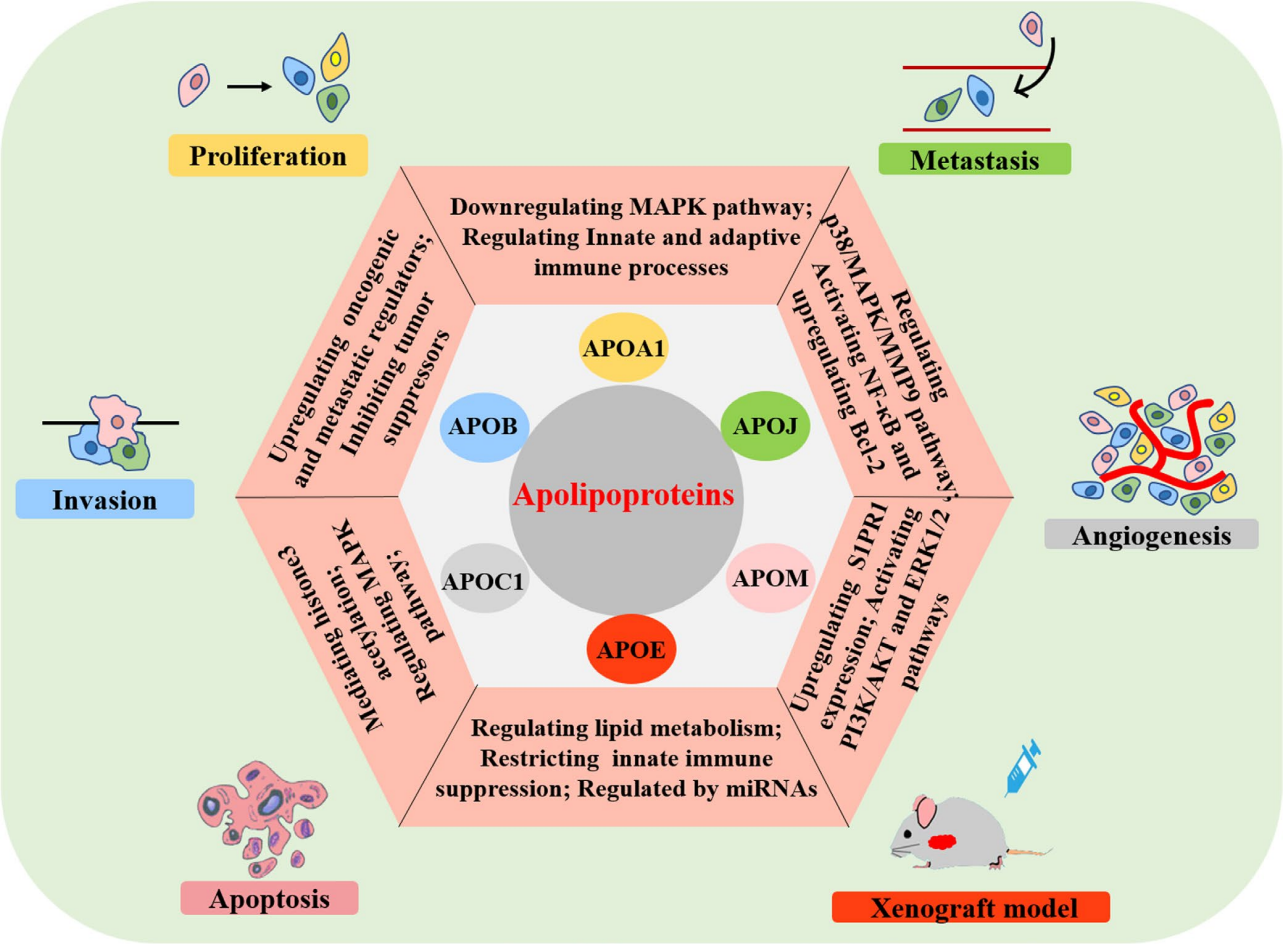
Functions and mechanisms of apolipoproteins as targets in cancers. All the functions and mechanisms of apolipoproteins which have been reported are described in this figure

Of the 22 APOs currently known (Table 1) most were found to have many vital functions in cancers via diverse mechanisms (Figure 2).

### 2.1 APOA and cancer

APOA1, APOA2, APOA4, and APOA5 are the main components of HDL particles.

The expression of APOA1 was reduced in some kinds of cancers while increased in others. The reduction in serum APOA1 levels was used as an independent predictor for metastasis or unfavorable prognosis of many cancers, such as ovarian cancer,^6^ nonsmall cell lung carcinoma (NSCLC),^7^ nasopharyngeal carcinoma (NPC),^8^ colorectal cancer,^9^ lymphoma,^10^ prostate cancer, 11 and renal cell cancer (RCC).^12^ On the other hand, increased expression of APOA1 was seen in some other types of cancers including small cell lung carcinoma (SCLC), hepatocellular carcinoma (HCC) and bladder cancer. Expression of APOA1 in SCLC was considerably higher than in normal controls and the presence of elevated levels correlated with the recurrence of SCLC.^13^ The concentration of APOA1 was higher in the serum of HCC patients,^14^ which could be an inferior prognostic.^15^ APOA1 was increased in urine from bladder cancer patients and could be considered a diagnostic marker,^16^ with low preoperative serum APOA1 levels predicting longer survival.^17^ The role of APOA1 in breast cancer has been controversial. Many studies showed that expression of APOA1 was inversely associated with development of breast cancer,^18^, ^19^, ^20^ but in a few studies, higher APOA1 expression was positively associated with promoting breast cancer.^21^

APOA1 mimetic peptides are 18‐amino acid sequences that recapitulate the secondary structure and partial function of APOA1.^22^ APOA1 mimetic peptides were found to inhibit the development of ovarian cancer,^23^ breast cancer,^24^ colon cancer,^25^ and pancreatic cancer 26 both in vitro and in vivo. APOA1 itself could also exert a suppressive effect on ovarian cancer.^23^ APOA1 might promote apoptosis and inhibit HCC cell proliferation by arresting the cell cycle via down‐regulation of the MAPK (mitogen‐activated protein kinase) pathway.^15^ APOA1 could suppress growth and metastasis of melanoma in vivo through both innate and adaptive immune pathways, but there were no significant direct suppressive effects by APOA1 on melanoma cells.^27^ Treatment with APOA1 mimetic peptides improved the phenotypic, inflammatory, and histopathological manifestations of colitis‐propelled carcinogenesis.^28^

The level of APOA2 in serum was dramatically reduced in patients with gastric cancer and multiple myeloma,^29^, ^30^ but increased in HCC and prostate cancer.^31^, ^32^ APOA2 was highly overexpressed in the cerebrospinal fluid of patients with pediatric brain tumor.^33^ Expression of APOA2 was significantly reduced in pancreatic cancer and APOA2 might be used as an early diagnostic marker and risk factor for it.^34^

APOA4 expression in HCC tissues was dramatically reduced compared to normal controls.^14^ The serum level of APOA4 was also reduced in the serum of patients with ovarian cancer.^35^

### 2.2 Apolipoprotein B and cancer

APOB was shown to regulate the expression of many genes in development of HCC and was related to poor prognosis in HCC patients.^36^ Low expression of APOB was related to the increase of metastatic and oncogenic regulators in HCC, such as FOXM1, MTIF, HGF, CD44, and ERBB2, and suppression of tumor suppressors, such as PTEN and TP53. Inactivation of APOB was linked to poor prognosis in HCC patients possibly through its function in regulating numerous genes concerned with the development of HCC.^36^

### 2.3 Apolipoprotein C and cancer

The APOC family consists of four members, APOC1, APOC2, APOC3, and APOC4, which are surface components of CM, VLDL, and HDL.^37^

APOC1 was overexpressed in pancreatic cancers and an increased level of APOC1 in preoperative serum of patients was considered to reflect an unfavorable prognosis. Knockdown of APOC1 expression inhibited proliferation and prompted apoptosis of pancreatic cancer cells.^38^ Overexpression of APOC1in breast cancer patients had diagnostic utility in distinguishing between triple‐negative breast cancer (TNBC) and non‐TNBC and thus was a potential prognostic factor for TNBC.^39^ APOC1 expression was increased in acute myeloid leukemia and played an oncogenic role in disease progression by mediating H3 acetylation regulated by ANP32A.^40^

Knockdown of APOC1 expression significantly suppressed proliferation of tumor cells and decreased colony formation, whereas overexpression of APOC1 increased growth of THP1 and HL60 cells. The expressions of APOC1 mRNA and protein were upregulated in prostate cancer tissues and the serum levels of APOC1 were increased in prostate cancer patients.^41^ The mRNA and protein of APOC1 were also highly expressed in lung cancer tissues at the late stage, but no prognostic effect of serum levels of APOC1 could be found in lung cancer patients.^42^

The serum levels of APOC1 were significantly decreased in NSCLC,^43^ colorectal cancer,^44^ papillary thyroid carcinoma 45 and child nephroblastoma,^46^ and might be a diagnostic or prognostic marker of these cancers. There were some evidences that APOC1 facilitated tumor progression in colorectal cancer through the MAPK signaling pathway.^47^

Serum levels of APOC2 were elevated in pancreatic cancer patients compared to controls and had prognostic value for surgery.^48^

### 2.4 Apolipoprotein D and cancer

APOD is an atypical apolipoprotein primarily associated with HDL in human plasma.^49^

APOD expression in HCC tissues was significantly lower than that in normal controls and it was identified as an independent prognostic marker of HCC.^50^ The mRNA expression of APOD was dramatically downregulated in colorectal tumors compared to normal colorectal tissues, and reduced expression of APOD was tightly related to lymph node metastasis status, advanced stages, and lower overall survival.^51^ The overall survival of patients with epithelial ovarian carcinoma was lower when tumors were APOD‐negative than in APOD‐positive tumors.^52^ APOD was identified as a biomarker for low grade, noninfiltrating primary CNS neoplasms.^53^ Low APOD expression was related to a shorter relapse‐free survival and poor prognosis in breast cancer.^54^

In contrast, APOD was highly expressed in malignant melanoma and might be useful as a prognostic marker of cutaneous malignant melanoma.^55^ Other evidence suggested that elevated cellular APOD expression correlated with malignant transformation of the prostate.^56^ The content of APOD was increased in the urine of patients with renal cell cancer.^57^

### 2.5 Apolipoprotein E and cancer

APOE consists of 299 amino acids with numerous amphipathic *α*‐helices. APOE has three main alleles: APOE‐ε2 (cys112, cys158), APOE‐ε3 (cys112, arg158), and APOE‐ε4 (arg112, arg158). These allelic forms differ from each other by two amino acids at positions 112 and 158, these differences alter APOE structure and function.^58^ Many kinds of cancers showed elevated expression of APOE.

Both APOE mRNA and protein levels were higher in NSCLC tissue^59^ and serum APOE was increased in NSCLC patients. Higher APOE levels correlated with lymph node metastasis, distant metastasis, TNM stages, and poor prognosis.^60^ APOE was up‐regulated in gastric cancer and such patients had shorter survival times. There was a strong link between APOE levels and risk of muscular invasion making it a promising marker for predicting the invasions of gastric tumors.^61^, ^62^

APOE was overexpressed in various ovarian cell lines and tissues and it was essential for growth and survival of ovarian cancer cells.^63^ The level of APOE in the serum of patients with ovarian cancer was dramatically increased over healthy individuals and as a marker, it could enhance the specificity and sensitivity of ovarian cancer diagnosis.^64^ APOE was highly expressed in the PC‐3 human prostate cancer cell line and its expression was directly correlated with the Gleason score of prostate cancer tissues, hormone independence and local and distant metastasis.^65^

APOE is among the best‐verified potential prognostic or diagnostic marker in many other cancers. Serum levels of APOE were related to the overall survival rate of metastatic colorectal cancer patients under chemotherapy and bevacizumab treatment.^66^ APOE was highly increased in the urine of bladder cancer patients and the levels correlated with the tumor stage.^67^ Thus, APOE testing of the urine could provide a potential marker for noninvasive bladder cancer.^68^ Increased levels of APOE were measured in the serum and tissues of pancreatic cancer patients and this may prove useful as an early screening tool for the disease.^69^ Higher serum levels of APOE were related to the progression of breast cancer and poor prognosis in the patients.^70^ APOE protein was frequently elevated in HCC tissues and might be a suitable histological marker for HCC.^71^

APOE participated in the transport of lipids to glioblastoma cells and in the recycling of lipids in necrotic areas by macrophages.^72^ Activation of APOE restricted the innate immune system's suppression of cancer cell proliferation, thus promoting tumor growth and metastasis in many types of cancers.^73^ APOE was regulated by various miRNAs and increased LRP1/LRP8‐dependent melanoma metastasis and angiogenesis.^74^

### 2.6 Apolipoprotein H and cancer

APOH is a multifunctional apolipoprotein encoded by the human APOH gene and one of its functions is to bind cardiolipin.^75^ APOH was highly overexpressed in hepatitis B‐related HCC tissue,^76^ significantly upregulated in the urine of renal carcinoma patients compared with healthy controls,^77^ and APOH expression was significantly increased in leukemia.^78^

### 2.7 Apolipoprotein L and cancer

APOL consists of APOL1, APOL2, APOL3, APOL4, APOL5, and APOL6, whose structures and functions are similar to those of the proteins of the Bcl‐2 family.^79^ APOL1 mRNA and protein expression was significantly elevated in the tissue of papillary thyroid carcinomas.^80^ Expression levels of APOL2 could predict the survival time of patients with bladder cancer.^81^ The APOL3 region on chromosome 22q12 was found to be a risk locus in hereditary prostate cancer.^82^ APOL6 was identified as homologous to a Bcl‐2 protein and could induce apoptosis mediated by mitochondria in cancer cells.^83^

### 2.8 Apolipoprotein M and cancer

There was higher expression of APOM in NSCLC tissues than in non‐NSCLS and of APOM overexpression promoted invasion and proliferation of NSCLC cells in vitro and tumor growth in vivo by upregulating expression of S1PR1 and activating the PI3K/AKT and ERK1/2 signaling pathways.^84^

In contrast, APOM mRNA and protein expression in HCC tissues were dramatically decreased compared to adjacent healthy tissues.^85^ Overexpression of APOM inhibited the proliferation, migration, and invasion of hepatoma cells and the development of xenograft tumors in nude mice, and promoted apoptosis.^86^ APOM mRNA and protein levels were notably reduced in colorectal cancer tissues, compared to adjacent healthy tissues, normal mucosa, polyps, and inflammatory mucosa.^87^

### 2.9 Apolipoprotein J and cancer

APOJ, also called clusterin, is a ubiquitous, secreted, 75‐80 kDa heterodimeric glycoprotein linked by disulfide bonds, which is involved in apoptosis and the clearance of cellular debris.^88^ It may be induced by stress and was identified as a cytoprotective chaperone protein that aids folding of secreted proteins. The three isoforms of APOJ were discovered to participate in pro‐ and antiapoptotic processes^89^ and were abnormally regulated in many severe physiological disturbances including cancer initiation and progression.^90^

Combined with other chemotherapeutic agents, antisense compounds targeting APOJ proved successful in clinical trials for the treatment of prostate cancer.^91^, ^92^ Expression of APOJ protein correlated with Gleason scores in prostate cancer.^93^ APOJ was also overexpressed in HCC tissues, which corresponded to higher TNM stages and inferior histological grade.^94^ Overexpression of APOJ promoted epithelial‐mesenchymal transition and migration of HCC in vitro and promoted metastasis in vivo.^95^ Silencing the APOJ gene enhanced the chemosensitivity of hepatic carcinoma cells.^96^

APOJ was highly up‐regulated in colon cancer and played oncogenic roles in multistage colorectal tumorigenesis, progression,^97^ and poor outcome.^98^ The level of APOJ in serum was significantly increased in colorectal carcinoma and could be used as a prediagnostic marker.^99^ The increased level of dissociative APOJ in highly aggressive tumors and metastatic nodes might be a predictive and prognostic marker for colon cancer aggressiveness.^100^

Overexpression of APOJ in ovarian cancer could be diagnostic^101^ and predictive of adverse outcomes.^102^ Levels of APOJ in plasma of ovarian cancer patients were abnormally elevated and might be used for early diagnosis of epithelial ovarian cancer.^103^ APOJ was significantly overexpressed in breast carcinoma^104^ and could be used as a prognostic factor,^105^ while blocking APOJ expression could inhibit the invasion and metastasis of human breast cancer cell lines.^106^

APOJ expression was significantly upregulated in RCC tissues and could be an independent prognostic factor.^107^ APOJ overexpression in gastric cancer was associated with tumor progression and metastasis^108^ and in pancreatic carcinoma with lymph node metastasis.^109^ However, cytoplasmic APOJ expression was related to longer survival in NSCLC patients after surgery.^110^

APOJ promoted metastasis of colon cancer^111^ and promoted invasion of tumor via the p38/MAPK/MMP9 pathway.^112^ APOJ also conferred resistance of breast cancer cells to TNF*α* and caused apoptosis via activation of NF‐*κ*B and overexpression of Bcl‐2.^113^

## 3 APOLIPOPROTEINS AND AUTOPHAGY IN CANCER

Autophagy is a cellular housekeeping process that degrades and recycles damaged organelles or misfolded proteins in lysosomes.^114^ It could limit inflammation and tumor necrosis, and mitigate DNA damage in tumor cells in response to metabolic stress.^115^ Autophagy exerts essential function in cancer metastasis^116^ and chemotherapy resistance,^117^ which provides a potential targeting strategy for the treatment of cancer.^118^

Autophagy is also involved in the homeostasis of lipids, regulating lipid stores, and promoting lipoprotein metabolism.^119^ Many studies have now shown that autophagy can promote the degradation of APOB.^120^, ^121^ Inhibition of APOB synthesis stimulated endoplasmic reticulum autophagy, which could prevent steatosis.^122^ APOE4 could inhibit autophagy gene products through direct binding to coordinated lysosomal expression and regulation (CLEAR) DNA motifs.^123^ APOL1, which specifically binds to BH3 (Bcl‐2 homology domain 3), could induce autophagic cell death through upregulating formation of autophagic vacuoles and triggering the translocation of LC3‐II (Autophagy‐Related Protein LC3‐II) from the cytosol to the vacuoles, when overexpressed and accumulated intracellularly.^124^ APOL6 could promote apoptosis and block beclin1‐dependent autophagy in atherosclerotic cells.^125^ Combination of APOA1‐modified liposome‐doxorubicin with autophagy inhibitors may overcome multidrug resistance in vitro.^126^

## 4 APOLIPOPROTEINS AND OXIDATIVE STRESS IN CANCER

Oxidative stress (OS) results when the balance is shifted between the systemic level of reactive oxygen species (ROS) and the detoxification of the reactive intermediates or repair of the resulting damage.^127^ The redox signaling pathways that respond to ROS, were often up‐regulated in various malignant tumors.^128^ Some researchers suggested that cancer metastasis was an adaptive approach for cancer cells to evade oxidative damage and escape from ROS.^129^

Knockdown of APOJ in human cancer cells suppressed cell proliferation, induced apoptosis, and significantly sensitized cells to both genotoxic and OS induced by chemotherapeutic drugs and H_2_O_2_.^90^ Expression of APOD was increased under OS in many pathological situations including cancers. One study found that APOD responded to OS in the tumor microenvironment and could serve as a marker of initial stages of tumor progression.^130^

## 5 APOLIPOPROTEINS AND DRUG RESISTANCE IN CANCER

APOA1 was reported to be associated with resistance to aromatase inhibitors in treatment of breast cancer^131^ and with resistance to carboplatin and paclitaxel, which are key chemotherapy drugs for epithelial ovarian cancer.^132^ It was reported that expression of APOD could be used as a novel biomarker of tamoxifen resistance in postmenopausal node‐positive breast cancer patients.^133^ Knockdown of APOE by siRNA reduced resistance of Hep3B cells to cardiac steroids through mediation of the Na+/K+‐ATPase signalosome.^134^

Inhibiting APOJ expression using antisense oligonucleotides enhanced sensitivity to androgens,^135^ chemotherapeutics,^136^ and radiation^137^ in prostate cancer. Down‐regulating APOJ gene expression could synergistically chemo‐sensitize bladder cancer cell lines and inhibit growth and metastasis of tumor cells both in vitro and in vivo.^138^ Suppression of APOJ expression inhibited the growth and metastasis in renal carcinoma models^139^ and enhanced the effect of cisplatin and sorafenib.^140^, ^141^ Increased APOJ expression could confer gemcitabine resistance in pancreatic cancer,^142^ while APOJ knockdown sensitized pancreatic cancer cells to gemcitabine.^143^ Knockdown of APOJ chemo‐sensitized human breast cancer cells both in vitro and in vivo.^113^ APOJ expression was correlated with paclitaxel resistance in cervical cancer cell lines, and resistance was dramatically decreased when the expression of APOJ was reduced by APOJ siRNAs in HeLaS3 cells.^144^ Expression of APOJ was increased in multidrug‐resistant osteosarcoma cells.^145^ Thus, APOJ may be used to predict the responsiveness of many cancers to chemotherapy.

## 6 CONCLUSIONS AND FUTURE PERSPECTIVES

Abundant evidence has suggested that the global expansion in excess body weight over the past several decades was closely associated with increasing cancer incidence,^146^, ^147^ suggesting a joint focus on lipid metabolism and related mechanisms in cancers. As we have attempted to illustrate above, recent data suggest that APOs participate in essential functions in various cancers.

**Figure 3:**
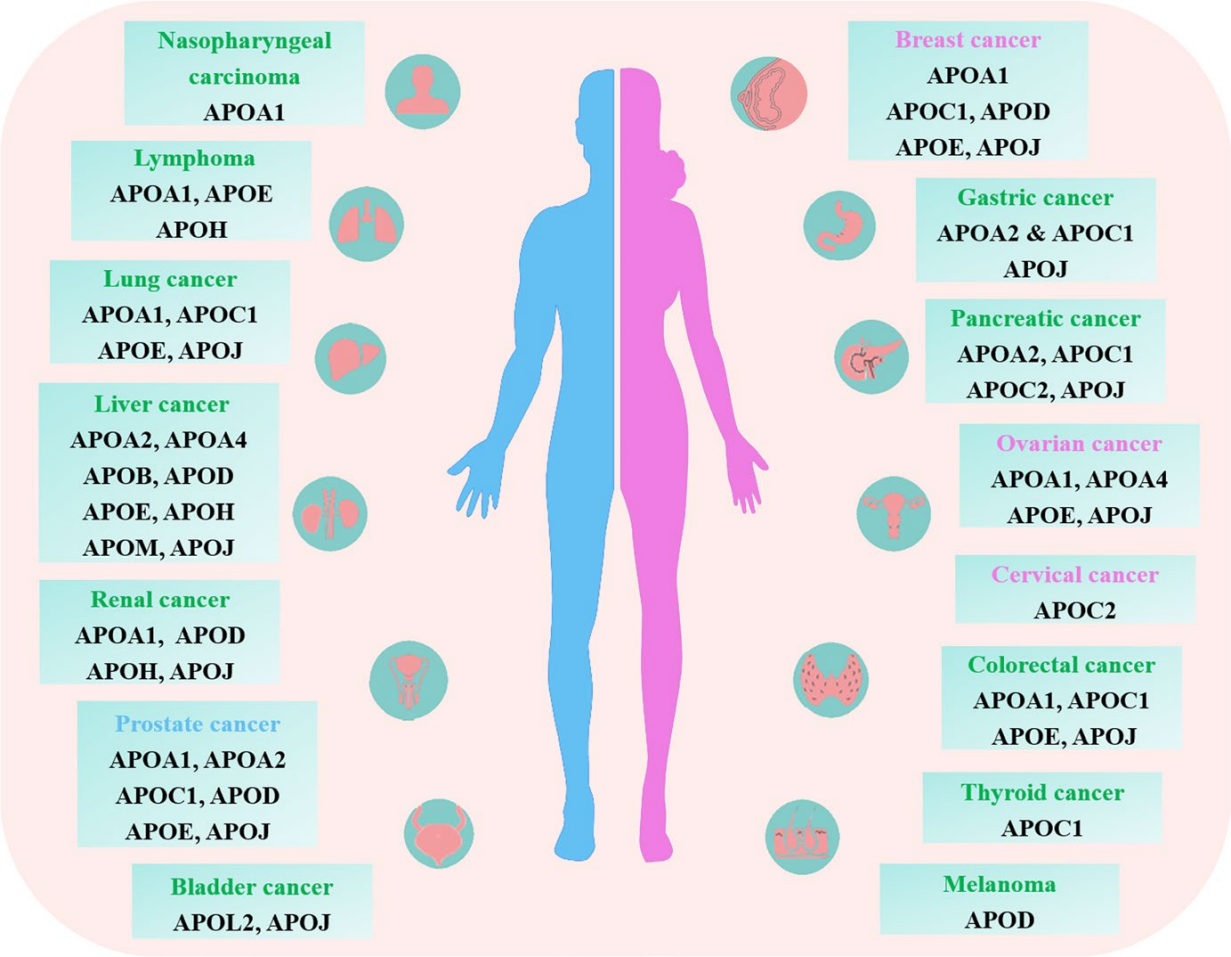
The biomarker landscape of apolipoproteins in cancers. All the relevant information about the application of apolipoproteins as biomarkers in cancers have been summarized in this figure

APOs are the protein part of plasma lipoprotein, which bind and transport blood lipids to various tissues of the body for metabolism and utilization.^2^ Many studies have found that mutated APOs with different allelic polymorphisms and phenotypes can result in abnormal blood lipid metabolism and utilization, thereby playing important roles in occurrence and development of hyperlipidemia, atherosclerosis, cardiovascular diseases and tumors. APOs could be useful for diagnosis and prognosis in cancer but also as potential therapeutic targets. However, some APOs can be abnormally expressed in different tumors, and there are aberrant expressions of different APOs in the same tumor (Figure 3). Combined screening for multiple APOs or using other methods to ameliorate the sensitivity and specificity of biomarkers in cancers may be the main research direction in the future. Several studies have produced contradictory results for APOs in some cancers, which may require further investigation with larger sample size and more rigorous experimental design.

Many APOs are overexpressed or downregulated in various tumor tissues and cells, and can potentially be used as therapeutic targets by inhibiting expression or using corresponding mimetic peptides. The most successful applications to date in the clinic are the APOJ antisense oligonucleotides for the treatment of prostate cancer. Besides, the APOA1 mimetic peptides have shown excellent therapeutic effects in different ovarian cancer models in vitro and in vivo and are promising candidates for further development.

The majority of APOs are being subjected to preclinical screening research and will necessitate further development. In addition, the expression of APOs is closely related to tumor sensitivity to chemotherapeutic drugs, and combining APO manipulation with drug treatment is expected to enhance their therapeutic effects. However, the mechanisms whereby APOs function in progression of cancers is still unclear, and more studies on APO regulation and metabolism are needed.

APOs are clearly promising therapeutic targets as well as useful diagnostic and prognostic biomarkers in cancers, but much further research is necessary to accelerate the clinical use of APOs and to enhance our understanding of their cancer‐related influences.

## ACKNOWLEDGMENTS

This work was funded by Beijing Natural Science Foundation (7172142) and by National Natural Science Foundation of China (81573454). This work was also funded by Technology Major Projects for “Major New Drugs Innovation and Development” (2018ZX09711001‐005‐025) and CAMS Innovation Fund for Medical Sciences (2016‐I2M‐3‐007).

## CONFLICT OF INTEREST

No conflicts of interest declared.
